# 

**DOI:** 10.1192/bjb.2022.44

**Published:** 2023-06

**Authors:** Dasal Abayaratne

**Affiliations:** Speciality trainee in medical psychotherapy and general adult psychiatry with Sheffield Health and Social Care NHS Foundation Trust, Sheffield, UK. Email: dasalabayaratne@googlemail.com

**Figure:**
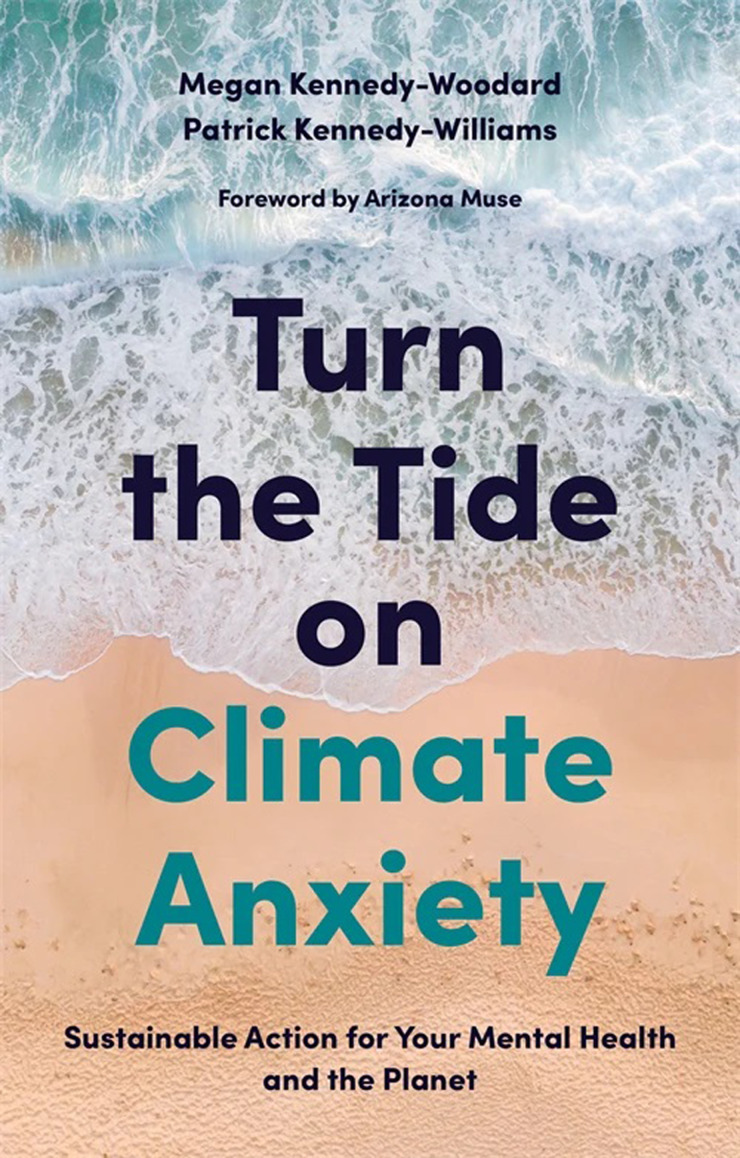

This book is one of many on climate action currently in the shops. However, it stands out from the pack. Instead of focusing on the ‘why’ of climate action, or even the ‘what’, the authors focus on the ‘how’. That is, they explore the emotional reactions climate change stirs up, including the psychological traps that may impede committed sustainable action, and how these can be overcome.

The opening section outlines a range of psychological reactions, including climate anxiety, depression and anger, and how ubiquitous these now are. Indeed, the authors clearly argue that although these may have an impact on our well-being, they are not pathological. Instead, they are an understandable reaction to a planet-sized threat. Perhaps it is even irrational not to be anxious?

The second half focuses on navigating these reactions. It asks us to observe and compassionately acknowledge our internal experiences. Following this, we learn about the psychological defences, cognitive biases and behavioural patterns that might unhelpfully hook us. To counter them, we are invited to connect with our values (not only environmentalism) and to act in line with them. Importantly, the authors add that this needs to be balanced with self-compassion to avoid personally unsustainable overactivity that inevitably leads to a harmful boom and bust. Instead, we are asked to be ‘okay with being okay’ (all positive actions are recognised, no matter how small) and to be ‘okay with not being okay’ (accepting that some things are beyond our control and responsibility).

Many psychological modalities, including acceptance and commitment therapy and psychoanalysis, are drawn on but rarely mentioned. Although this may disappoint some, it makes for an accessible read that is not bogged down in theory. The book is further brought to life through interactivity and real-world vignettes. Crucially, this is not a textbook, rather a living, breathing guide that invites the reader to connect with the material through personal reflection and exercises.

Despite already working in mental health and climate change, through reading this book, I found myself actively making changes to my own life and in a better place to help others, including those in clinic. The book, however, is not just for those already in the field, or for those affected by climate anxiety, but for anyone. We are all touched by climate change in some way.

